# Supplementary material for: Proteomic Analysis of Low-Grade, Early-Stage Endometrial Carcinoma Reveals New Dysregulated Pathways Associated with Cell Death and Cell Signaling
Source: Cancers (Basel). 2021 Feb 14;13(4):794. doi: 10.3390/cancers13040794 (PMC7917913; doi:10.3390/cancers13040794)
Supplement: Supplementary file 1 [file cancers-13-00794-s001.zip › cancers-1059925 - supple/Supplementary Figures and Tables.docx]

Supplementary Materials:

Proteomic Analysis of Low-Grade, Early-Stage Endometrial Carcinoma Reveals New Dysregulated Pathways Associated with Cell Death and Cell Signaling

Álvaro López-Janeiro, Ignacio Ruz-Caracuel, Jorge L. Ramón-Patino, Vivian De Los Ríos, María Villalba Esparza, Alberto Berjón, Laura Yébenes, Alicia Hernandez, Ivan Masetto, Ece Kadioglu, Virginie Goubert, Victoria Heredia-Soto, Rodrigo Barderas, José Ignacio Casal, Carlos E. de Andrea, Andrés Redondo, Marta Mendiola, Alberto Peláez-García and David Hardisson


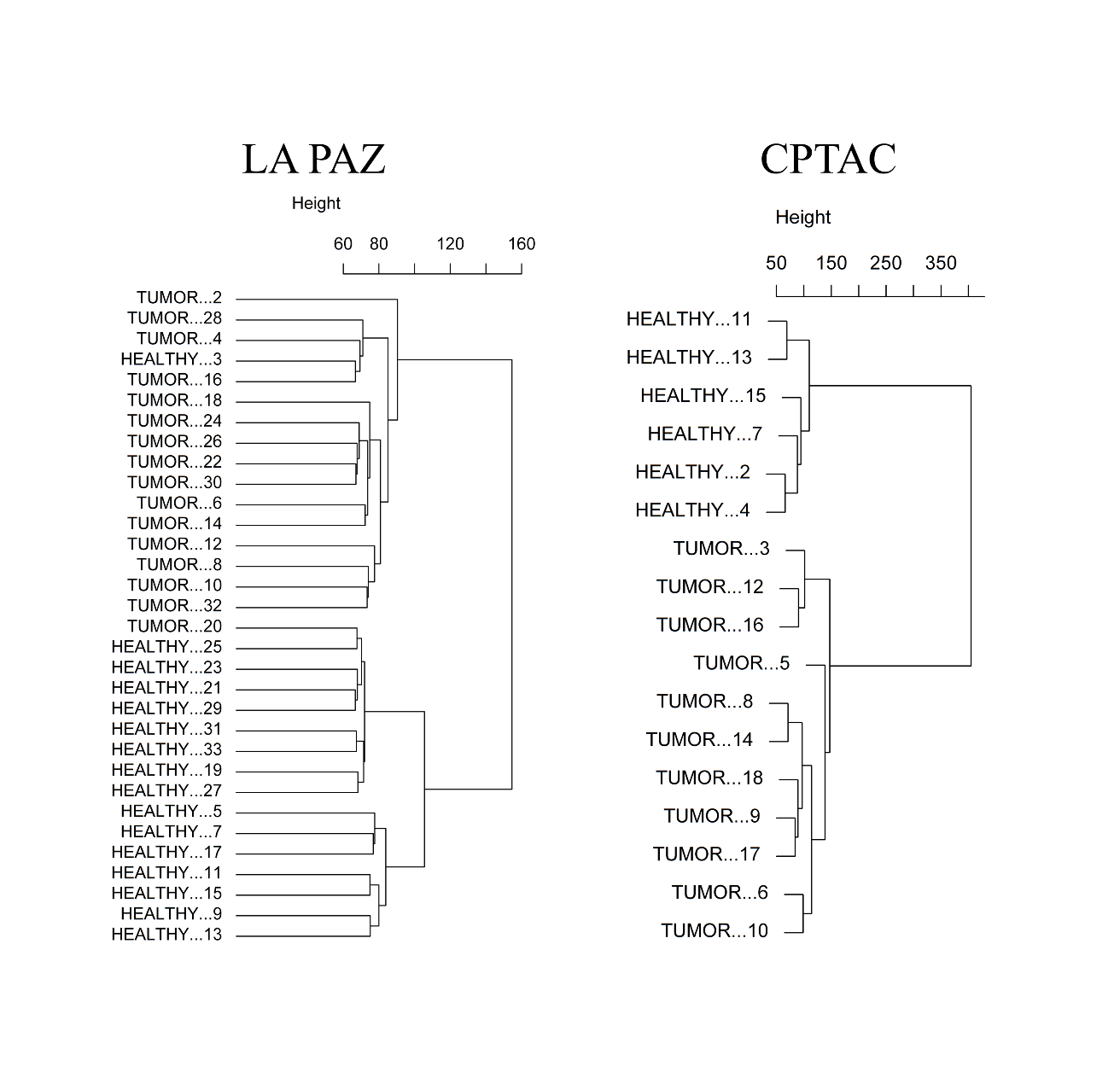


**Figure S1.** Hierarchical clustering for the discovery and validation cohort.


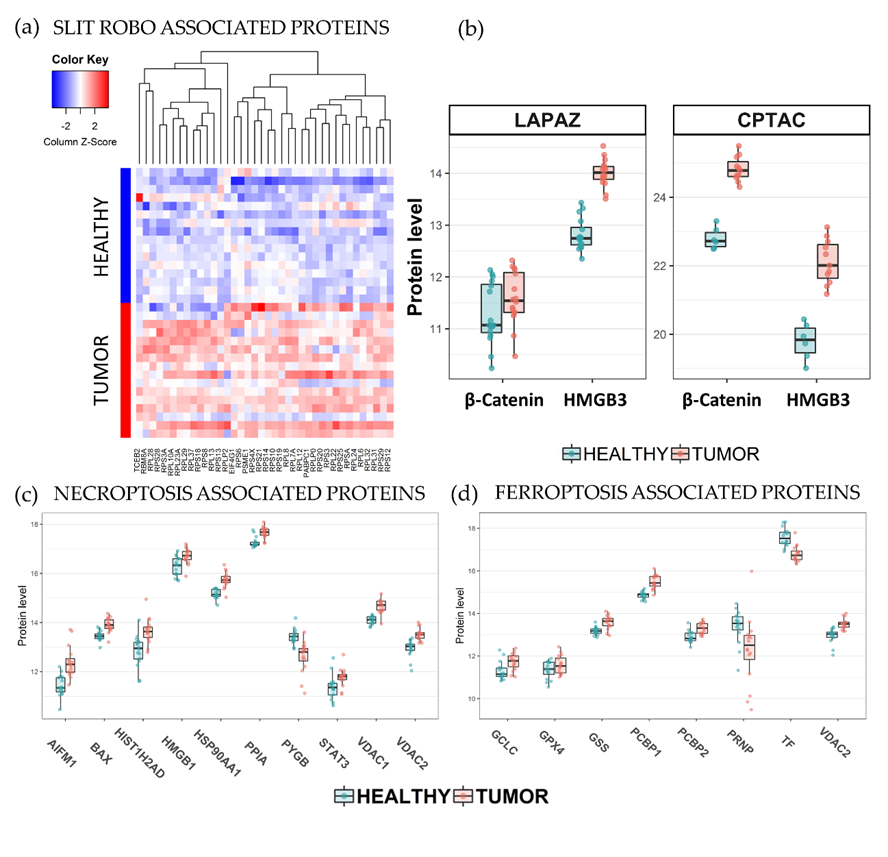


**Figure S2.** (a): Heatmap representing SLIT/ROBO pathway associated protein expression in the discovery set. Samples are arranged according to Healthy/Tumor status. (b): HMGB3 and β-catenin protein expression in the discovery and validation set according to proteomic data. (c): Boxplots representing Necroptosis pathway associated protein expression in the discovery set. (d): Boxplots representing Ferroptosis pathway associated protein expression in the discovery set.


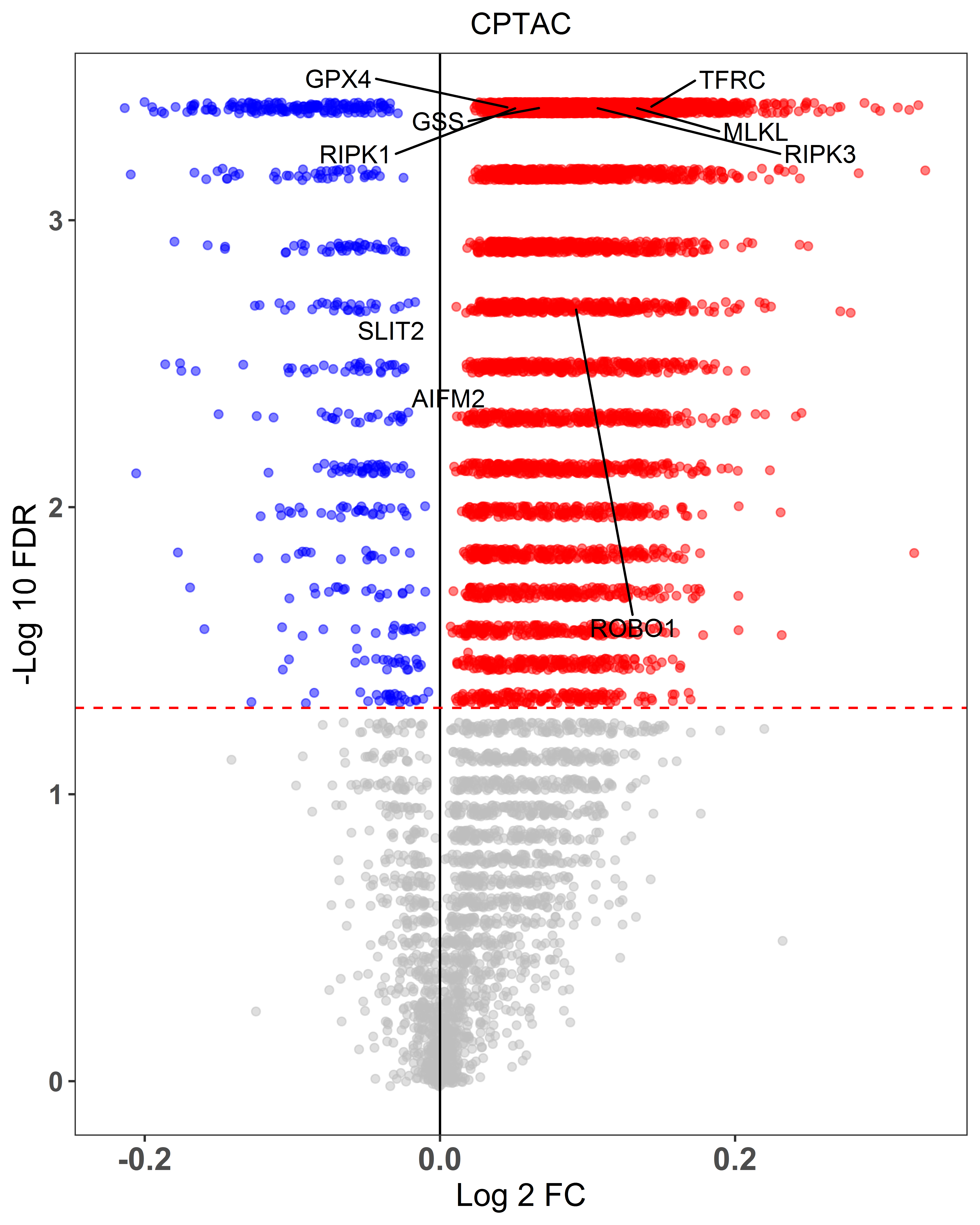


**Figure S3.** Volcano plot depicting differentially expressed proteins in the validation Set. Key proteins associated with SLIT/ROBO, Ferroptosis and Necroptosis pathways are pointed out.


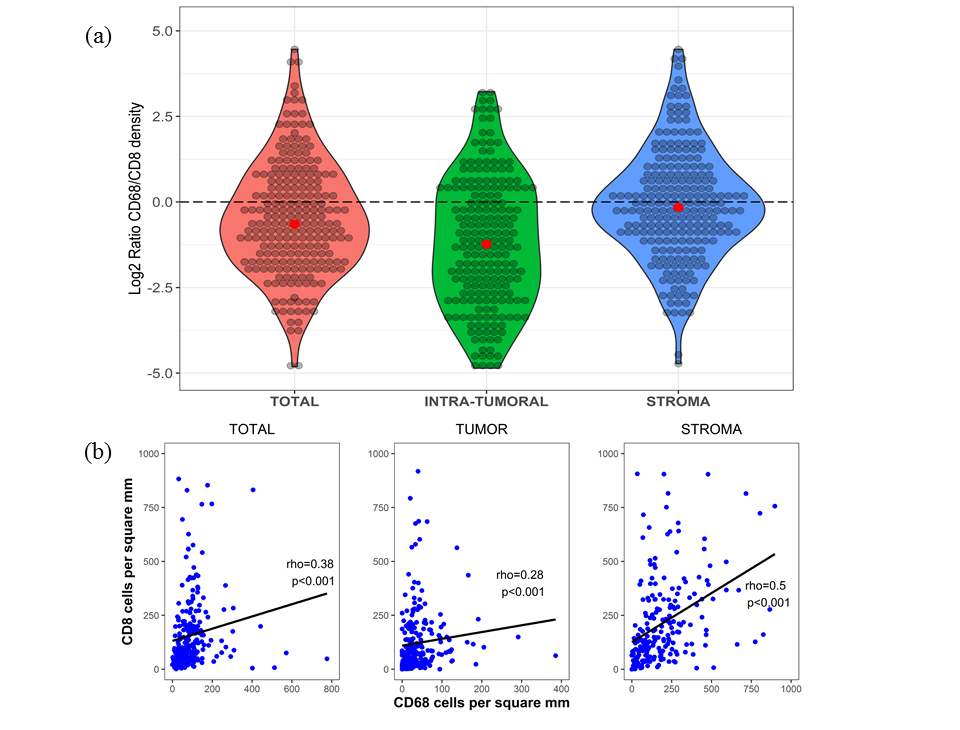


**Figure S4.** Violin plots of CD68/CD8 ratios and correlation between cell densities.

**Table S2:** Clinicopathological variables of the discovery cohort.

| **Dicovery cohort clinicopathological variables** | |
| --- | --- |
| **CLINICAL VARIABLES** |  |
| **Age at diagnosis (median IQR)** | 73.5 (66.5 - 78) |
| **Nº of patients with relapsed disease (n, %)**  **(10 years follow up)** | 8/16 (50%) |
| **Pattern of relapse** | Local = 2/8 (25%)  Lymph Node = 4/8 (50%)  Distant = 6/8 (75%) |
| **Relapse Free Survival (months, median IQR)** | 61.8 (19.2 - 82.93) |
| **PATHOLOGICAL VARIABLES** | |
| **Grade (n, %)** | Grade 1 = 10/16 (62.5%)  Grade 2 = 6/16 (37.5%) |
| **FIGO stage at Diagnosis (n, %)** | FIGO IA = 5/16 (31.2%)  FIGO IB = 11/16 (68.8%) |
| **Lymphovascular invasion (n,%)** | Absent = 12/16 (75%)  Present = 4/16 (25%) |
| **Presence of differentiation** | Squamous = 7/16 (43.7%)  Mucinous = 5/16 (31.1%) |
| **Mismatch protein status** | Conserved = 8/16 (50%)  MLH1/PMS2 loss = 5/16 (31.2%)  MSH2/MSH6 loss = 3/16 (18.8%) |

**Table S3.** List of primers used for transcriptomic validation.

| **Transcript** | **Sense** | **Sequence 5'-3'** |
| --- | --- | --- |
| SLIT-2 | Forward | CCAGCTTCTGCCATTTACAGTGT |
|  | Reverse | TTCCCTGGCATGCCTCCTA |
| ROBO2 | Forward | TGGAGACCTCACAATCACCA |
|  | Reverse | GGCTGGGCCTTGTAGAATTA |
| HMGB3 | Forward | GACCAGCTAAGGGAGGCAA |
|  | Reverse | ACAGGAAGAATCCAGACGGT |
| CFLAR | Forward | CCTCACCGACGAGTCTCAAC |
|  | Reverse | CCGTGGTCCTTGTTGTCTCA |
| PPIA | Forward | GAGGAAAACCGTGTACTATTAGC |
|  | Reverse | TTGTCTGCAAACAGCTCA |
| GAPDH | Forward | GGAGCGAGATCCCTCCAAAAT |
|  | Reverse | GGCTGTTGTCATACTTCTCATGG |


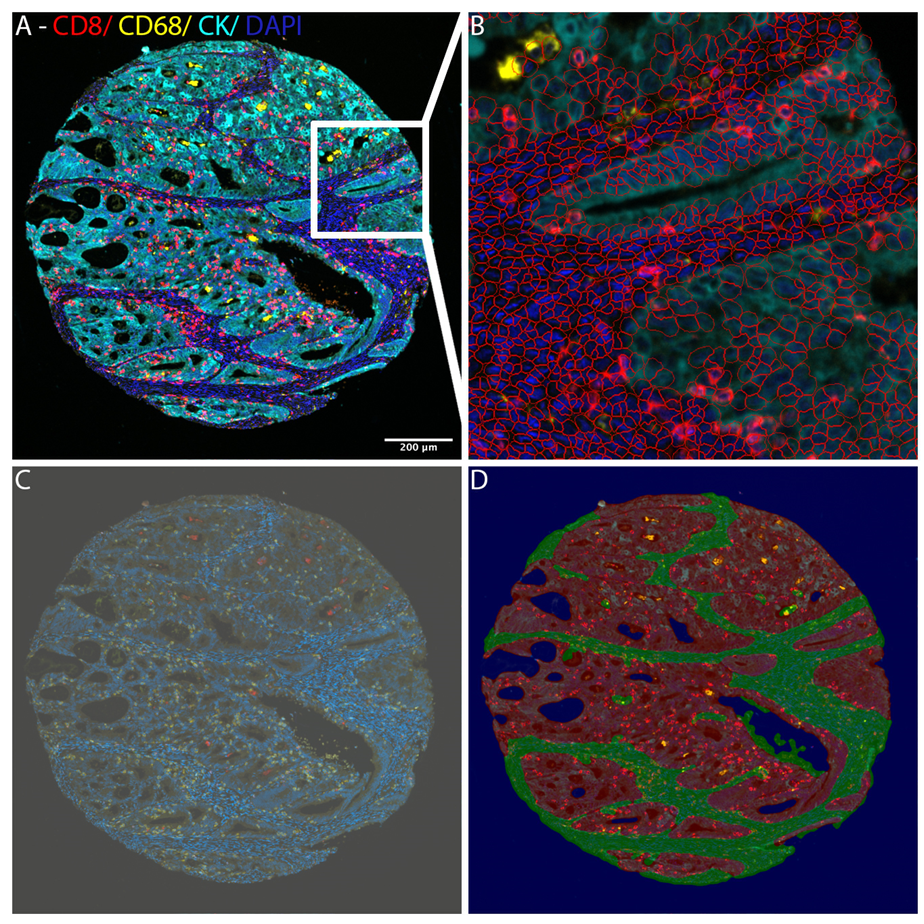


**Figure S5.** Representative images of cell segmentation and phenotyping process (A-C) and tissue segmentation algorithm (D).
